# Effects of Postural Interventions on Physical and Psychological Aspects of Children in Terms of Secondary Sexual Characteristics

**DOI:** 10.3390/ijerph19127401

**Published:** 2022-06-16

**Authors:** Maki Maekawa

**Affiliations:** Department of Physical Education, International Pacific University, 721 Kanonji, Seto-cho, Higashi-ku, Okayama 709-0863, Japan; m.maekawa@ipu-japan.ac.jp

**Keywords:** posture, alignment, physical and psychological health, junior high school students

## Abstract

Children with secondary sexual characteristics who experience considerable physical, psychological, and social development are prone to physical and mental imbalances. The effects of postural intervention on physical and psychological aspects in junior high school students in terms of secondary sexual characteristics were investigated in this study. Of the 30 participants in this study, 21 (10 boys, 11 girls) with complete measurements were included. The postural intervention consisted of 1 month of direct muscle stretching for postural maintenance and breathing as well as activities to orient the spinal curvature. The participants’ body arrangement, spinal curvature, and General Health Questionnaire 30 (GHQ) scores were compared before and after the postural intervention. The intervention improved postural alignment (e.g., head–neck angle *t*_20_ = 2.33, *p* < 0.05, 95%CI [0.30, 5.36]) and GHQ scores (e.g., GHQ total *t*_20_ = 3.36, *p* < 0.01, 95%CI [0.79, 3.40]). The postural intervention improved the posture of the students as well as positively affected their mental health. This study showed that students with secondary sexual characteristics can receive physical and psychological care without the use of special facilities or techniques.

## 1. Introduction

Posture and health are closely related; moreover, various symptoms result from postural disorders. Previous studies have reported that improper posture in children at school and in everyday life might impact their postural development [1,2,3]. Moreover, the musculoskeletal system can be damaged by movements associated with improper posture, thereby restricting one’s ability to perform daily activities and diminishing one’s quality of life [4,5,6].

The human spine forms a natural physiological curve in proper posture, thereby exhibiting an alignment known as “cervical and lumbar lordosis” and “thoracic and sacral kyphosis” [6,7]. In the sagittal plane, a proper physiological standing posture shows a straight line through the mastoid process–acromion–hip joint, which is slightly in front of the knee joint and the lateral malleolus [8]. Such a posture is reportedly more energy-efficient because it ensures stability with less load on the muscles, skeleton, and nerves [6,8]. However, poor posture is generally defined as a rounded back or a posture with a pronounced forward position of the acromion and head [8,9]. Excessive loading of joint structures and muscular tension caused by deviation from the ideal postural arrangement can result in functional and structural pathologies (e.g., headaches, neck and shoulder pain, and degenerative intervertebral discs) [10,11,12,13]. Furthermore, previous studies have revealed that improper posture in children and adolescents might result in the severe problems mentioned above in late adulthood [2,10,14]. It is thus essential to develop proper posture from childhood itself.

Especially in children in the secondary sexual characteristics period, muscle growth is slower than bone growth, resulting in a relative shortening of muscles and tendons, which are constantly susceptible to tension. Therefore, muscular and skeletal imbalances and changes in body size may negatively affect postural maintenance during these periods. A 5-year cross-sectional study on developmental changes in static posture in middle and high school students reported that the head and shoulder positions tend to be more forward from the eighth grade through the first year of high school [15]. Moreover, during the secondary sexual characteristics period, it is well known that the secretion of hormones increases; the physical development of nerves, muscles, and bones as well as emotional, intellectual, and social developments are remarkable [16,17,18]. Posture and the physical and psychological states are linked and impact one another [19,20]. In previous research that examined postural interventions’ psychological effects, the authors conducted a postural intervention on high school students who experienced school truancy and found that the intervention positively affected the students’ mental health and that they felt better adjusted to school as their posture improved [21]. Harvey [22] also reported that college students who received postural feedback training showed improvements in subjective posture and on the 36-Item Short Form Health Survey questionnaire on physical function, emotions, energy/fatigue, confidence, and overall stress ratings. Therefore, postural adjustment may be an effective approach to improve mental health.

Recently, there has been an increase in the use of information and communication technology terminals, such as personal computers and tablets, in schools and at home, and children’s posture is highly vulnerable to disruption. In addition, children’s mental health is a pressing issue. According to the State of the World’s Children 2021 report, more than 13% of adolescents aged 10–19 years, including 80 million adolescents aged 10–14 years and 86 million adolescents aged 15–19 years, are estimated to live with a diagnosed mental disorder based on the definition of the World Health Organization. Among the diagnosed mental disorders, anxiety and depression account for approximately 40%, whereas the other diagnoses include attention deficit/hyperactivity disorder, conduct disorder, intellectual disability, bipolar disorder, eating disorders, autism, schizophrenia, and personality disorders [23].

Therefore, maintaining and caring for children’s physical and psychological health during their secondary sexual characteristics period is extremely important because this has an effect not only on their physical and psychological development but also on their present and future well-being. In this study, the effects of a postural intervention on physical and psychological aspects were investigated in junior high school students during the period of secondary sexual characteristics.

## 2. Materials and Methods

### 2.1. Participants

In total, 30 students in middle school who belonged to a track and field club participated in the study. Of these, 21 subjects (10 boys and 11 girls; mean age: 13.3 ± 1.1 years, height: 159.4 ± 4.8 cm, weight: 47.5 ± 4.8 kg) who participated in all measurements were included in the analysis. Informed consent was obtained from the participants and their parents after providing them with a description of the experimental protocol, which was approved by the ethics committee at the International Pacific University (approval no: 2017-1).

### 2.2. Procedure

This study was based on a single-group pretest/posttest design. The intervention period was approximately 1 month, and measurement data included both pre- and post-intervention data, including postural measurement items and subjective questionnaires on physical and psychological health.

#### 2.2.1. Postural Alignment Measurement during Usual Standing

Participants were barefoot and wore light clothing. The marker was attached to the left side of their bodies at the auricular lobule, acromion, midpoint of the greater trochanter, lateral condyle of the femur, and lateral malleolus. They were then instructed to maintain their usual standing posture, with both feet 10 cm apart, and to gaze at the same markers in front of them at eye level. The image of the standing posture of their whole body was taken in the sagittal plane.

#### 2.2.2. Measurement of Spinal Curvatures

The spinal curvatures and pelvic tilt in the sagittal plane were measured using the Spinal Mouse System (Idiag, Fehraltorf, Switzerland), which is a noninvasive measuring device that allows computing the angle between each vertebra, by moving the measuring sensor from the spinous process of C7 to that of the S3. These landmarks were first determined by palpation. Subsequently, the distance and angle between the vertebrae were measured by tracing the spinous processes of the spine using two rolling wheels on a spinal mouse. These data were transferred from the device to a personal computer, which was sampled at every 1.3 mm distance as the mouse rolled along the spine, with a sampling frequency of approximately 150 Hz.

The sum of the angles between each vertebra from T1–T2 to T11–T12 was defined as the thoracic kyphosis angle and that from T12–L1 to the sacrum was defined as the lumbar lordosis angle. The angle between the straight line connecting the S1 and S3 and the vertical line was defined as the sacral inclination angle, whereas that between the straight line connecting the T1 and S1 and the vertical line was defined as the trunk inclination angle.

Participants were instructed to maintain their usual standing posture, stay barefoot with feet 10 cm apart, and look at a fixed point at eye level. The participants were then measured in three test positions: (1) standing posture (in a usual standing position with both hands naturally hanging by the sides of the body), (2) maximal flexion (bending the body as far as comfortably possible from a usual standing position so that the head is on the knees and the arms are extended to the floor or at the toes), and (3) maximal extension (tilting the trunk backward from the usual standing posture and extending it as far as comfortably possible).

#### 2.2.3. Questionnaire Survey on Physical and Psychological Conditions

The Japanese version of the General Health Questionnaire (GHQ) 30 was used to assess subjective physical and psychological status. This questionnaire was designed for children and adults aged ≥ 12 years and is a self-report instrument with 30 items, including the following six subscales: general illness, somatic symptoms, sleep disturbance, social dysfunction, anxiety and dysphoria, and depression. Each subscale has five items, and a total score was calculated for both the subscale (range, 0–5) and overall general health scores (range, 0–25). The Japanese version of the GHQ has already been evaluated for reliability and validity [24].

### 2.3. Postural Intervention

First, this study focused on the diaphragm and scalene muscles, which are involved in postural maintenance and breathing movements, and pressure was applied to these muscles with fingers to increase their flexibility. Finger pressure was applied to the diaphragm using index–little fingers for approximately 5 s while exhaling, and the pressure was then released while inhaling. This sequence of actions was repeated five times. Similarly, finger pressure was applied to scalene muscles for approximately 5 s using the index fingers while inhaling, and the pressure was then released while exhaling. This sequence of actions was repeated five times. Subsequently, actions to orient the spinal curvature were performed. To orient the curvature of the cervical vertebrae, participants maintained a fixed shoulder position, moved the head forward, smoothly backward flexed the head as if staring at the ceiling, and slowly moved the head with the second cervical vertebra as the fulcrum such that the face turned forward. Participants maintained a standing posture with the pelvis slightly tilted forward and the chest and abdomen extended; they bent slightly forward with the hip joints as a fulcrum, and slowly raised the upper body to orient the thoracic and lumbar curvature. Subsequently, the neutral position of the pelvis was set by applying force to the lower abdomen. Finally, the posture was maintained with both scapulae slightly adducted and the chest expanded [21]. All participants were instructed to perform these actions for postural improvement once per day at least once every 2–3 days, for approximately 1 month. These actions were easy to complete in 1–2 min any time of the day such as during club activities or at home.

### 2.4. Data Analysis

To evaluate the postural alignment in the sagittal plane, the ideal was defined as a vertical line passing 3 cm anterior to the lateral malleolus; the distances between the auricular, acromion, greater trochanter, and knee joint marker points and the ideal line were calculated and presented as absolute values. The head–neck angle was calculated as the angle between the straight line connecting the auricle and acromion and the vertical line. To evaluate spinal curvature, thoracic kyphosis, lumbar lordosis, sacral inclination, and trunk inclination angles were used. Postural alignment in the usual standing position was compared with ideal alignment, and flexibility was evaluated by the trunk tilt angle at maximum flexion and extension. A positive calculated value indicated kyphosis/forward tilt and a negative calculated value indicated lordosis/backward tilt.

The subscale score, which is the total score for each GHQ item, was calculated during the psychological survey evaluation; thereafter, the overall psychological health score was calculated by totaling all GHQ items. Score values are indicated on a scale of 0–25, with higher values indicating more intense symptoms.

These postural (body alignment and spinal curvature) and psychological (each GHQ component point) measures were compared before and after the intervention.

### 2.5. Statistical Analysis

The Shapiro–Wilk test was performed to test all data for normality, and paired *t*-test was used to evaluate normally distributed data. The probability of significance (*p*), 95% confidence intervals, and the effect size (Cohen’s dz) to examine the extent of the difference were calculated to evaluate the difference between the pre-test and post-test means of each indicator. Cohen’s dz was calculated as the mean difference between pre- and post-test divided by the standard deviation of the mean difference [25]. Pearson’s correlation coefficient was used to examine the relationship between the changes in the postural and psychological states associated with the intervention. The risk ratio was set at less than 5%. All statistics were obtained using SPSS Statistics 26 (IBM Japan, Tokyo, Japan).

## 3. Results

Table 1 shows the mean values and standard deviations of the postural measurement parameters pre- and post-intervention. After the intervention, the body alignment of all measured sites approached a more ideal alignment (auricular lobule, *t*_20_ = 4.75, *p* < 0.001; acromion, *t*_20_ = 3.94, *p* < 0.001; midpoint of the greater trochanter, *t*_20_ = 3.85, *p* < 0.001; lateral condyle of the femur, *t*_20_ = 3.53, *p* < 0.01). The head–neck angle was significantly smaller after the intervention (*t*_20_ = 2.33, *p* < 0.05), as was the thoracic kyphosis angle and trunk inclination angle (thoracic kyphosis, *t*_20_ = 3.55, *p* < 0.01; trunk, *t*_20_ = 1.85, *p* = 0.08). After the intervention, the lumbar lordosis angle and the sacral inclination angle in the usual standing position were significantly greater (lumbar lordosis, *t*_20_ = 4.26, *p* < 0.001; sacral, *t*_20_ = −5.82, *p* < 0.001). The range of maximal flexion and extension was significantly greater after the postural intervention (flexion, *t*_20_ = −2.12, *p* < 0.05; extension, *t*_20_ = 5.69, *p* < 0.001).

Table 2 shows the means and standard deviations of the GHQ scores pre- and post-intervention. General illness (*t*_20_ = 2.59, *p* < 0.05), anxiety and dysphoria (*t*_20_ = 4.93, *p* < 0.001), depression (*t*_20_ = 2.25, *p* < 0.05), and total GHQ scores (*t*_20_ = 3.36, *p* < 0.01) decreased significantly.

**Figure 1:**
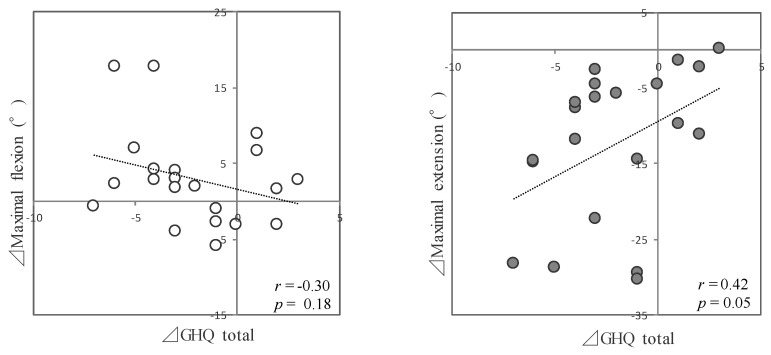
Correlation between the changes in the GQH total scores and flexibility.

Correlations were found between posture and psychological measures (trunk inclination and sleep disturbance: *r* = 0.33, *p* = 0.14; maximal flexion and depression: *r* = −0.35, *p* = 0.12; maximal flexion and GHQ total: *r* = −0.30, *p* = 0.18; maximal extension and anxiety and dysphoria: *r* = 0.33, *p* = 0.14; maximal extension and depression: *r* = 0.41, *p* = 0.06; and maximal extension and GHQ total: *r* = 0.42, *p* = 0.05) (Figure 1).

## 4. Discussion

This study aimed to evaluate standing posture and investigate the effects of the postural intervention on the physical and psychological aspects of junior high school students in terms of secondary sexual characteristics. The results show that after the intervention, the trunk inclination in the standing position became more vertical and increased the flexibility of the maximal flexion and extension. Moreover, many subjects showed better scores on the GHQ, a subjective measure of physical and psychological health, which also improved after the postural intervention.

In proper posture, the human spine forms a natural physiological curvature, which curves forward in the lumbar spine and backward in the thoracic spine [6,7]. In the sagittal plane, a proper physiological standing posture shows a straight line through the mastoid process–acromion–hip joint, which is slightly in front of the knee joint and the lateral malleolus [8]. The postural features of the participants in this study were a forward head protrusion (81.0%) and an anterior (50.0%) and posterior (27.3%) tilt of the trunk during usual standing. The postural intervention in this study brought the head and trunk tilt to a vertical position and each body part to an ideal body alignment. In the posture with the head forward, the activity of the extensor muscle groups, including the trapezius, increases to keep the head up. According to a previous study, the greater the head tilt, the greater the load on the neck [26]. In addition, forward and backward bending place more load on the spine and the intervertebral disc than the upright posture [27,28]. In the present study, the head position was placed or brought closer to directly above the trunk the and tilt of the trunk was brought closer to vertical, which is believed to reduce the strain on the muscle of the neck and lower back, and tendon organization.

Static muscle stretching has been demonstrated to reduce muscle stiffness and hardness in studies [29,30]. Finger pressure was used in this study to reduce stiffness in the diaphragm and scalene muscle, both of which are critical for maintaining posture and breathing. The diaphragm contributes to postural stability by increasing intra-abdominal pressure. Furthermore, diaphragmatic activity during breathing motions comprises thoracic and abdominal expansion movements that occur with inhalation, as well as simultaneous changes in intra-abdominal pressure and thoracic motion. Thus, increased flexibility of the diaphragm is considered to affect postural stability and ease of breathing movements. Additionally, the scalene muscle is related to the anterior flexion and lateral rotation of the head and to the expansion of the upper thorax by pulling up the first and second ribs. The increased flexibility of the scalene muscle can facilitate breathing and allow the head to move easily in anterior–posterior flexion and rotation. Thus, in this study, stretching of the diaphragm and scalene muscles is believed to be interrelated and results in increased flexibility, easier posture maintenance, and easier breathing. Notably, this study revealed that the postural intervention brought the participants’ posture closer to the ideal alignment; however, further research is required on the relationship between posture and breathing.

It should also be noted that after the posture intervention in this study, there was a remarkable improvement in not only posture but also psychological aspects and competition results. The main content of the GHQ consists of questions about whether normal psychological functioning was maintained or whether new events that were distressing the subject occurred. Higher GHQ scores indicate greater symptom severity, and the scores are generally proportional to severity. In the present study, the items of general illness, anxiety and dysphoria, depression, and total GHQ scores showed significant decreases after the intervention, indicating a positive effect of the postural intervention on physical and psychological health status. Additionally, correlations were found between postural and psychological measures. It is possible that after the intervention, the increased flexibility and changes in head and trunk postural maintenance style reduced physical stress and facilitated postural maintenance and breathing, as indicated by the GHQ’s physical and psychological health status.

This study revealed that adjusting posture not only improves objective physical aspects of the body but also has a positive impact on subjective physical and psychological health. Postural interventions are a valuable approach for supporting and developing students’ physical and psychological health during the development of secondary sexual characteristics.

The changes in mental and physical status with intervention observed in the present suggest the presence of some effects on daily activities. It is interesting to note that most of the students who participated in track and field recordings showed improved athletic performance after only one month of postural intervention. Movement is represented as a continuous change in posture. The motor function has both postural and motor aspects. Although these two aspects are differentiated functional systems to a certain extent, coordinated temporal and spatial movement of posture and movement is essential for congruent voluntary movement. Therefore, posture and movement are not controlled separately but are considered to be expressed as a closely controlled dynamic resting state. The present study results suggest that improvement in the usual standing posture leads to a change in the motor style of running and throwing to a more rational and congruent style of locomotion; however, more detailed examination is a topic for future study.

The present study had several limitations that should be acknowledged. The identification of the measurement points by palpation and the measurement of spinal curvature was always performed by the same person, thus making it impossible to show interobserver differences. While this study was carefully performed by an examiner skilled in measurement, to reduce human error, it should be considered that measurements and assessments be performed as concentrated and meticulous as possible. The study included a single group that was evaluated before and after intervention, and the small number of measurers and time constraints hindered the inclusion of a larger number of students in the study. Future studies to investigate the effects of postural intervention in more detail will include more participants and a control group which will be provided with equivalent postural education after the study period. In addition, the ease of postural maintenance, breathing, and efficient activity of the musculoskeletal and respiratory circulatory systems associated with postural interventions was assumed to be reflected in psychological aspects; however, no data were obtained to provide evidence for these factors. Further studies on the effects of interventions during static posture on physical function and the evaluation of dynamic posture are needed.

## 5. Conclusions

This study evaluated usual standing posture and examined the effects of a postural intervention on the physical and psychological states of junior high school students during the period of secondary sexual characteristics. The results showed that the intervention improved the students’ posture as well as had a positive effect on their mental health. This study demonstrates that physical and psychological care for students during the period of secondary sexual characteristics can be provided without the use of special facilities or techniques.

## Figures and Tables

**Table 1:** Means and standard deviations of postural measurement parameters pre-and post-intervention.

	Dependent Variables	Pre Intervention		Post Intervention		Statistical Values	Singificance	95%Cl of the Difference	Cohen’s dz
		Mean	(SD)	Mean	(SD)			(Lower, Upper)	
Standing	Head-neck (°)	11.0	(8.5)	8.2	(6.2)	*t*_20_ = 2.33	*p* < 0.05	0.30, 5.36	0.51
	Auricular lobule (cm)	6.3	(3.0)	3.9	(2.4)	*t*_20_ = 4.75	*p* < 0.001	1.36, 3.48	1.04
	Acromion (cm)	4.5	(2.8)	2.3	(2.2)	*t*_20_ = 3.94	*p* < 0.001	1.02, 3.32	0.86
	Greater trochanter (cm)	5.2	(2.6)	2.8	(1.9)	*t*_20_ = 3.85	*p* < 0.001	1.12, 3.76	0.84
	Lateral condyle (cm)	2.7	(2.3)	1.6	(1.9)	*t*_20_ = 3.53	*p* < 0.01	0.44, 1.71	0.77
	Thoracic kyphosis (°)	37.0	(8.8)	31.1	(11.5)	*t*_20_ = 3.55	*p* < 0.01	2.44, 9.39	0.77
	Lumbar lordosis (°)	−25.1	(6.5)	−31.2	(7.2)	*t*_20_ = 4.26	*p* < 0.001	3.08, 9.00	0.93
	Sacral inclination (°)	14.1	(4.3)	20.3	(4.3)	*t*_20_ = −5.82	*p* < 0.001	−8.44, −3.98	−1.27
	Trunk inclination (°)	1.8	(1.5)	1.2	(1.0)	*t*_20_ = 1.85	*p =* 0.08	−0.08, 1.37	0.40
Maximal flexion	Trunk inclination (°)	113.6	(22.8)	116.5	(22.2)	*t*_20_ = −2.12	*p* < 0.05	−5.71, −0.04	−0.46
Maximal extension	Trunk inclination (°)	−38.0	(10.8)	−50.4	(9.7)	*t*_20_ = 5.69	*p* < 0.001	7.85, 16.93	1.24

Abbreviations: SD, standard deviation.

**Table 2:** Means and standard deviations of GHQ scores pre- and post-intervention.

	Dependent Variables	Pre Intervention		Post Intervention		Statistical Values	Singificance	95%Cl of the Difference	Cohen’s dz
		Mean	(SD)	Mean	(SD)			(Lower, Upper)	
GHQ	General illness	1.4	(1.2)	0.9	(1.2)	*t*_20_ = 2.59	*p* < 0.05	0.10, 0.95	0.57
	Somatic symptoms	1.3	(1.2)	1.5	(1.2)		N.S	−0.66, 0.28	
	Sleep disturbance	1.0	(1.1)	1.0	(1.2)		N.S	−0.52, 0.61	
	Social dysfunction	1.1	(1.8)	1.0	(1.7)		N.S	−0.22, 0.41	
	Anxiety and dysphoria	2.1	(1.6)	1.0	(1.5)	*t*_20_ = 4.93	*p* < 0.001	0.60, 1.49	1.08
	Depression	1.1	(1.8)	0.5	(1.1)	*t*_20_ = 2.25	*p* < 0.05	0.04, 1.10	0.49
	GHQ total	8.0	(6.0)	5.9	(5.4)	*t*_20_ = 3.36	*p* < 0.01	0.79, 3.40	0.73

Abbreviations: GHQ, General Health Questionnaire 30; SD, standard deviation.

## Data Availability

The data presented in this study are available on request from the corresponding author. The data are not publicly available because of legal and privacy issues.
